# Hexane soluble extract of *Mallotus philippensis* (Lam.) Muell. Arg. root possesses anti-leukaemic activity

**DOI:** 10.1186/1752-153X-7-157

**Published:** 2013-09-17

**Authors:** Musa Khan, Rizwana Aleem Qureshi, Masroor Hussain, Khalid Mehmood, Rahmat Ali Khan

**Affiliations:** 1 Department of Plant Sciences, Quaid-i-Azam University Islamabad, Islamabad, Pakistan; 2 Department of Pharmacognosy, Faculty of Life Sciences, University of Vienna, Althanstrasse 14, Vienna, Austria; 3 National Engineering and Scientific Commission (NESCOM), Rawalpindi, Pakistan; 4 Khyber Medical University, Peshawar, Pakistan; 5 Department of Pharmacy, Hazara University Havelian Campus, Abbottabad, Pakistan; 6 Department of Biotechnology, University of Science and Technology Bannu, Bannu, Pakistan; 7 Department of Biotechnology, Faculty of Biological Sciences, University of Science and Technology Bannu, Khyber Pakutunkhwa, Pakistan

**Keywords:** *Mallotus philippensis*, HL-60 cells, GC-MS, Cdc25A, Cyclin D1

## Abstract

**Background:**

*Mallotus philippensis* (Lam.) Muell. Arg. is a well known medicinal plant of Asia and Australia. Various compounds from different aerial parts of the plant have been reported possessing potent pharmacological, antiviral, antibacterial and cytotoxic activities. We were interested to determine the effects of some root extracts from *M. philippensis* on human promyelocytic leukemia HL-60 cell proliferation, cell cycle regulators and apoptosis in order to investigate its anti-leukemic potential.

**Results:**

Root extract of *M. philippensis* was initially extracted in organic solvents, hexane, ethyl acetate, and n-butanol. The hexane extract showed highest toxicity against p53-deficient HL-60 cells (IC_50_ 1.5 mg dry roots equivalent/ml medium) after 72 h and interestingly, inhibition of cell proliferation was preceded by the upregulation of the proto-oncogenes Cdc25A and cyclin D1 within 24 h. The hexane extract induced 18% apoptosis after 48 h of treatment. Chemical composition of the hexane extract was analyzed by GC-MS and the 90% fragments were matched with polyphenolic compounds.

**Conclusions:**

The present study confirms that the hexane fraction of *M. philippensis* root extract possesses anti-leukemic activity in HL-60 cells. The polyphenols were the main compounds of the hexane extract that inhibited proliferation and induced apoptosis.

## Background

*Mallotus philippensis* (Lam.) Muell. Arg (Euphorbiaceae) are shrubs or small trees which grow on mountain slopes or valleys, limestone hills or river valleys and forests at an altitude of 300–1600 m in Asia and Australia. Different parts of the plant have been used in traditional medicine. Kamala, a red powder consisting of glandular hairs from plant capsule has been used as anthelmintic and cathartic in traditional medicine [1,2] and an orange dye for silk [3]. Kamala is commonly administered in its curd form for the elimination of intestinal worms and also for skin irritation, ringworm, and freckles [4]. The fruit of the plant is purgative for animals [5].

The pericarp of *M. japonicus* exhibit cytotoxic and anti-tumour effects [6-8]. *M. philippensis* possesses anti-allergic properties [9] and is bactericidal for chemoresistant *Helicobacter pylori* strains [10]. Other species of *Mallotus* contain human immunodeficiency virus (HIV) reverse transcriptase inhibitory activities [11], anthelmintic and antibacterial activities [12,13]. The stem bark of *M. philippensis* contains 3 α-hydroxy-D:a-friedoolean-an-2-one that is toxic for tumour cells [14]. Here we investigated the effects of *M. philippensis* root extracts on the leukemic cells.

## Materials and methods

### Cell culture

HL-60 human promyelocytic cells were obtained from the American Type Culture Collection (Manassas, VA, USA). Cells were grown in RPMI 1640 medium supplemented with 10% heat inactivated fetal calf serum, 1% L-Glutamine and 1% Penicillin/Streptomycin (Life Technologies, Paisley, Scotland) at 37°C in a humidified atmosphere containing 5% CO2.

### Collection and extraction of root powder

*Mallotus philippensis* was collected from Margalla Hills (Islamabad, Pakistan) and identified by one of the authors in the Herbarium of Quaid-i-Azam University Islamabad. Roots were washed, air dried and grounded. Powdered *M. philippensis* root (20 g) was extracted four times (each for hexane, ethyl acetate and n-butanol) with methanol (MeOH). These extracts were collected and concentrated with a Rotavapor at 40°C. The three separate concentrated MeOH extracts were dissolved in distilled water and extracted three times each with hexane, ethyl acetate (EtOAc), n-butanol (BuOH) and concentrated to complete dryness. 9.23 g, 4.00 g and 7.08 g dried hexane, EtOAc and BuOH extracts were obtained respectively.

### Gas chromatography and mass spectrometry

Active hexane soluble fraction of *M. philippensis* root was determined qualitatively using a gas chromatography system that was interfaced with an Agilent 5973 inert mass selective detector (MSD) system (Wilmingto, USA). Other specifications were; Column: DB-5 MS; 30 m × 0.25 mm × 0.5 μm (Agilent J&W DB-5 ms Ultra Inert); Mode: Electron ionization (EI) scan mode; Mass range scanned: 25–800 amu; Source temperature: 230°C; Scan time: 0–60 min; Transfer line temperature: 280°C; Mass data processed software: Agilent Chemstation. Gas chromatography conditions were: Agilent 5890 N GC system; Injection mode: Split mode 10:1; Injection temperature: 250°C; Injection volume: 1 μl; Carrier gas: Helium; Flow rate: 1.5 mL/min; Oven temperature: 120-300°C.

### Growth inhibition assay

HL-60 cells were seeded in T-25 tissue culture flasks (Life Technologies, Paisley, Scotland) at a concentration of 1 × 10^5^/ml and incubated with increasing concentrations of different extracts of *M. philippensis*. Cell counts and IC_50_ values were determined in different fractions after 48 and 72 h, using a KX 21 N microcell counter (Sysmex, Kobe, Japan).

### Hoechst dye 33258 and propidium iodide double staining

Hoechst staining was performed according to method described by Grusch *et al.*[15]. HL-60 cells (0.1 × 10^6^/ml) were seeded in T25 cell culture flasks and exposed to increasing concentrations of *M. philippensis* hexane extract for 48 h. Hoechst 33258 (HO) and propidium iodide (PI, both Sigma, St Louis, MO) were added directly to the cells to final concentrations of 5 and 2 mg/ml, respectively. After incubation for 60 min at 37°C, cells were examined under fluorescence microscope (Axiovert, Zeiss) equipped with a filter and a camera. This method allows discriminating between early apoptosis, late apoptosis, and necrosis. Cells were judged according to their morphology and the integrity of their cell membranes, which could easily be observed after PI staining.

### Western blotting

HL-60 cells were reinsulated for increasing time periods (from 2 to 48 h) with 1.5 mg dry roots equivalent/ml medium extract/ml. Then, cells were placed on ice, washed with ice-cold PBS (pH 7.2), centrifuged (1000 rpm, 4°C, 4 min) and the pellets lysed in 150 μl buffer containing 150 mM NaCl, 50 mM Tris pH 8.0, 1% Triton X-100, 2.5% 0.5 mM PMSF and PIC (Sigma, Schnelldorf, Germany). Debris was removed by centrifugation (12,000 rpm, 4°C, 20 min) and the supernatant collected. Then equal amount of protein was loaded onto 10% polyacrylamide gels. Proteins were electrophoresed for 2 h and then electroblotted onto PVDF membranes (Amersham, Buckinghamshire, UK) at 4°C for 1 h. To confirm equal sample loading, membranes were stained with Poinceau S. After washing with TBS (Tris base, NaCl, PH 7.6 adjust with HCl), membranes were blocked for 1 h in blocking solution containing 5% skimmed milk in TBS and 0.5% Tween 20, washed 3 times in TBS/T, and incubated by gentle rocking with primary antibodies (cyclin D1 (Signaling (Danvers, MA, USA), Cdc25A (F-6) and Cdc25A (M-191) from Santa Cruz (Santa Cruz, CA, USA), β-Actine from Sigma (St. Louis, MO), in Blotto (0.2-0.3 : 1000) at 4°C overnight. Membranes were washed in TBS/T (3 × for 5 min) and further incubated with secondary antibody (peroxidase conjugated anti-rabbit IgG, or anti-mouse IgG) diluted to 1:2000 in Blotto, for 1 h at room temperature. Membranes were washed with TBS/T and the chemoluminescence was detected by exposure of the membranes to Amersham HyperfilmTM ECL (Amersham, Buckinghamshire, UK). The antibody against Cdc 25A (F-6) was purchased from Santa Cruz Biotechnology (Santa Cruz, CA, USA) and against cyclin D1 was from Cell Signaling (Danvers, MA, USA) and against β-actin was from Sigma (St. Louis, MO, USA).

### Cell cycle distribution analysis

HL-60 cells (0.5 × 10^6^ per ml) were seeded in T-25 tissue culture flasks and incubated with 1.5 mg dry roots equivalent/ml medium extract/ml. After 24 h, the cells were harvested and suspended in 5 ml cold PBS, centrifuged (600 rpm, 5 min), resuspended and fixed in 3 ml cold ethanol (70%) for 30 min at 4°C. After two washing steps in cold PBS, RNAse A and PI were added to a final concentration of 50 mg/ml each and incubated at 4°C for 60 min before analyses. Cells were analyzed with a Calibur flow cytometer (BD Biosciences, San Jose, CA, USA) and cell cycle distribution was calculated with ModFit LT software (Verity Software House, Topsham, ME, USA).

### Statistical analyses

To test the effect of extract triplicates of biological and technical repeats were used. The results of apoptosis and proliferation experiments were analyzed with *t*-test using GraphPad Prism version 4 (GraphPad Prim Sofware, Inc., San Diego, CA, USA).

## Results and discussion

### Inhibition of HL-60 cell proliferation by *M. philippensis* extracts

**Figure 1:**
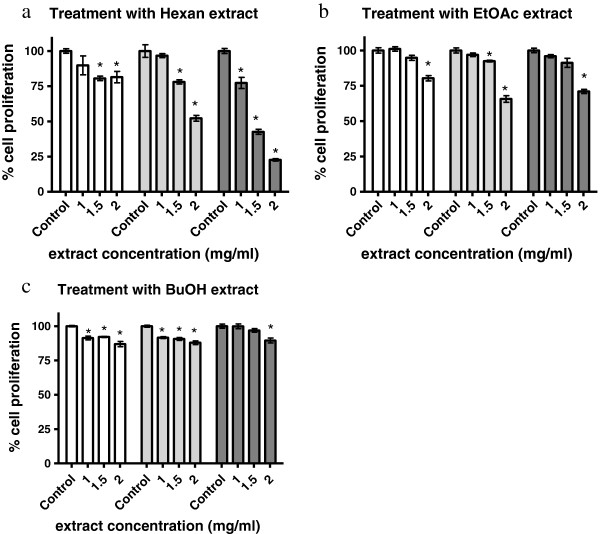
**Anti-proliferative effect of *Mallotus phillipensis *extracts.** HL-60 cells were seeded into T-25 tissue culture flasks (1 × 10^5^ cells/ml), grown for 24 h to enter logarithmic growth phase, and incubated with increasing concentrations **(a)** hexane extract (1.00, 1.50, and 2.00 mg dry roots equivalent/ml medium); **(b)** EtOAc extract (1.00, 1.50 and 2.0 mg dry roots equivalent/ml medium); **(c)** BuOH extract (1.00, 1.5 and 2.0 mg dry roots equivalent/ml). Cells were counted after 24, 48 and 72 h of treatment (white, light gray and dark gray columns, respectively) and the percentage of proliferation was calculated and compared to DMSO-controls (Control). Controls were considered as cells with a maximal proliferation rate (100%). Experiments were done in triplicate. Error bars indicate SEM, asterisks significance (p < 0.05).

Logarithmically growing HL-60 cells were incubated with increasing concentrations of n-hexane, EtOAc and BuOH extract for 72 h. Then, cells were counted and the inhibition of proliferation was calculated. The hexane extract showed highest toxicity against HL-60 cells (IC_50_ 1.5 mg dry roots equivalent/ml medium) after 72 h (Figure 1). The inhibition of HL-60 proliferation that was observed upon treatment with hexane extract was preceded by the down regulation of the proto-oncogene cyclin D1 after 48 h. Suppression of cyclin D1 is potent mechanisms to block cancer cell growth.

### Effect of hexane extract on cell cycle distribution

**Figure 2:**
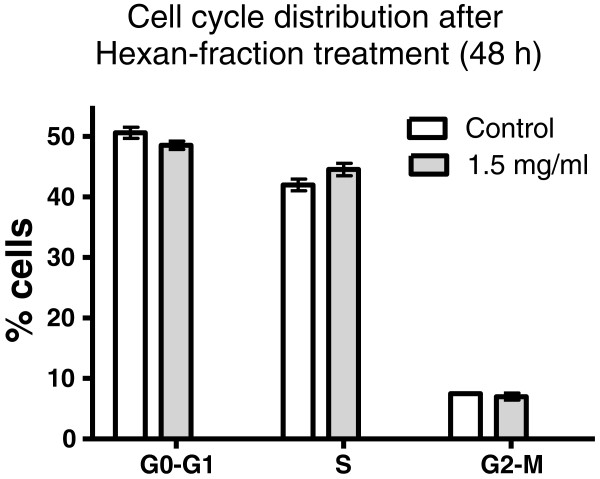
**Cell Cycle Distribution of HL-60 cells upon treatment with hexane extract of *Mallotus philippensis *for 48 h.** Logarithmically growing HL-60 cells were incubated with 1.5 mg dry roots equivalent/ml medium then subjected to FACS analysis. Experiments were done in triplicate. Error bars indicate SEM, (p > 0.05).

**Figure 3:**
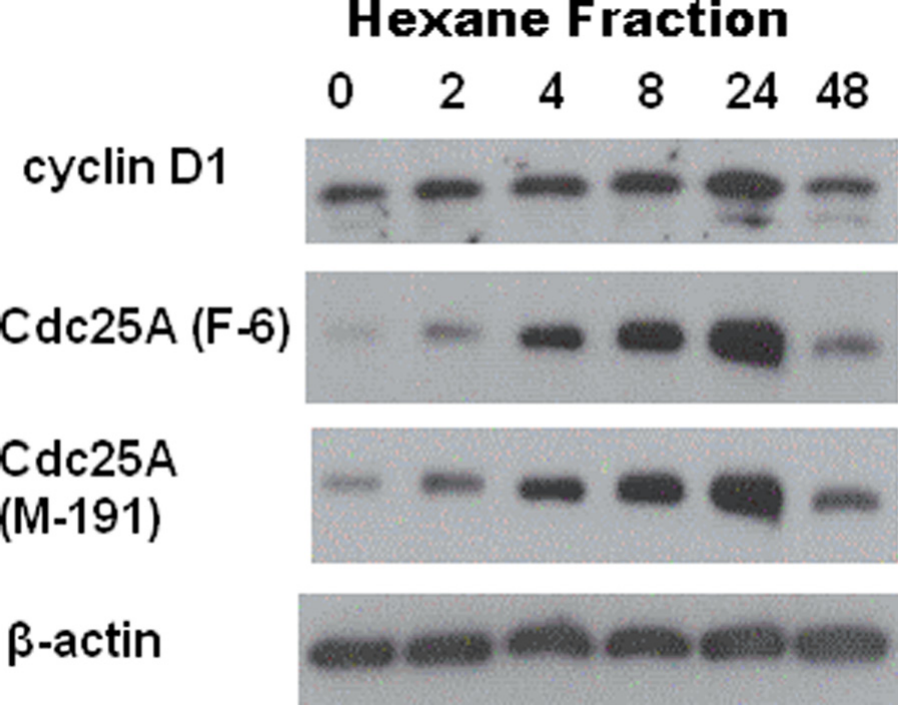
**Induction of cell cycle regulators by the hexane extract.** HL-60 cells (1 × 10^6^ cells) were seeded into T-25 tissue culture flasks and allowed to grow for 48 h when cells were incubated with 1.5 mg dry roots equivalent/ml medium extract/ml and for 2, 4, 8, 24 and 48 h. Then, isolated protein samples were subjected to 10% SDS-PAGE separation and subsequent Western blot analysis using the indicated antibodies. Equal sample loading was controlled by Poinceau S staining and β-actin analysis.

HL-60 cells were exposed to 1.5 mg dry roots equivalent/ml medium hexane fraction for 48 h to investigate cell cycle distribution. An accumulation of HL-60 cells in S-phase at the expense of G1-phase cells was observed, which however, was not significant (Figure 2). The increased number of S-phase cells suggested that the cell cycle became induced and this was substantiated by the increased expression of cyclin D1 and Cdc25A within 24 hours of extract treatment (Figure 3). Whereas cyclin D1 expression returned to control level after 48 hours, the expression of Cdc25A was still over expressed compared to control. Therefore, the expression of these two proto-oncogenes could not explain the inhibition of HL-60 proliferation and hence P value is worthless for the experiment. This implicated that other mechanisms blocked cell division.

### Induction of apoptosis by the hexane extract of *M. phillipensis*

**Figure 4:**
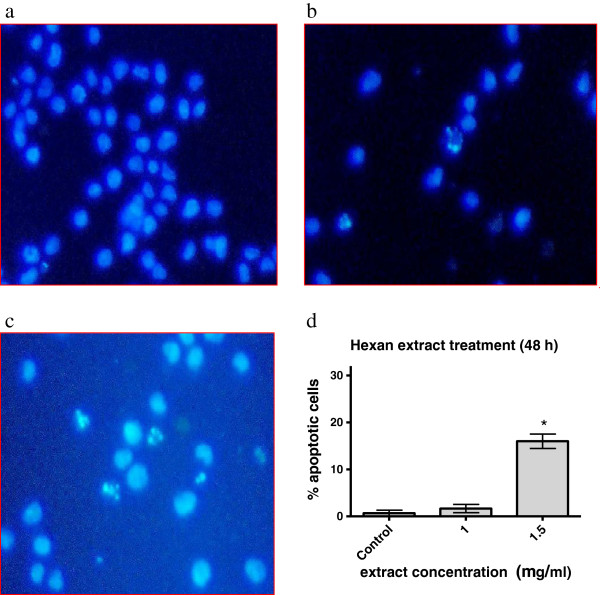
**Induction of apoptosis by the *Mallotus philippensis *hexane extract.** HL-60 cells were incubated with increasing extract concentrations (1 and 1.5 mg/ml dry root equivalent) for 48 h. Then, cells were double stained with Hoechst 33258 and propidium iodide, and examined under a fluorescence microscope with DAPI filter. Nuclei with morphological changes indicating apoptosis (Methods) were counted. **(a)**. control; **(b)**. 1 mg dry roots equivalent/ml medium and **(c)**. 1.5 mg dry roots equivalent/ml medium. The percentages of vital and apoptotic cells calculated **(d)**. Experiments were done in triplicate. Error bars indicate SEM, asterisks significance (p < 0.05).

The upregulation of oncogenes or upstream cell cycle protagonists together with the inhibition of downstream effectors of cell division tend to generate apoptotic stimuli. Therefore, logarithmically growing cells were incubated with increasing concentrations of the hexane extract of *M. philippensis* (1 and 1.5 mg dry roots equivalent/ml medium) for 48 h and the induction of cell death was analyzed. The extract induced apoptosis in 18% after 48 h of treatment (Figure 4).

### GC-MS Analysis of the hexane fraction of *M. philippensis*

**Figure 5:**
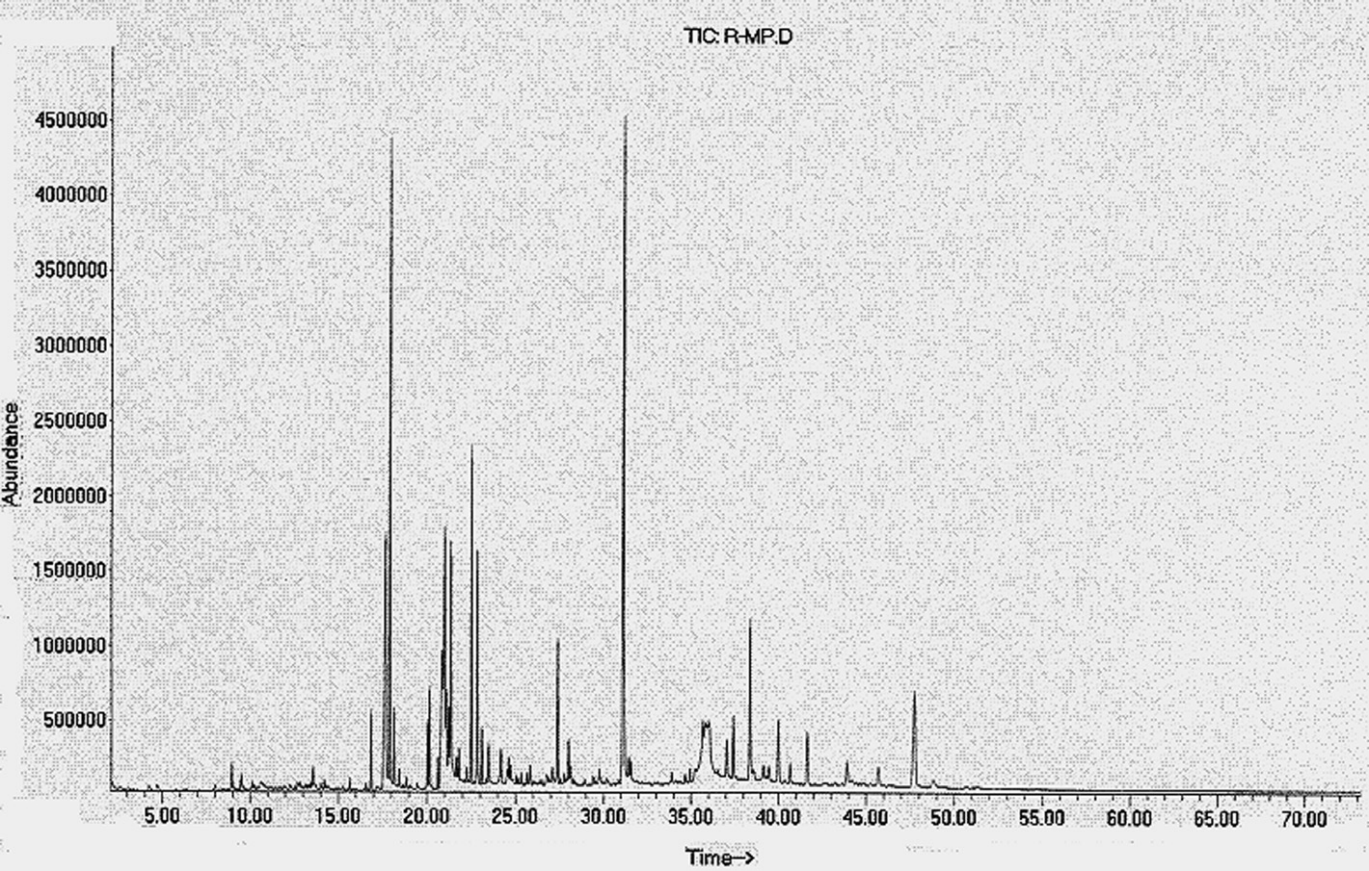
**GC chromatogram of hexane soluble fraction of *Mallotus philippensis*.**

GC-MS analyses of *M. philippensis* hexane fraction were performed to identify the volatile and semi volatile components (Figure 5). Several suspected polyphenolic compounds (GC R_f_ = 17.917, 31.125, 39.9, 45.66, 43.905 and 47.735 minutes) have been detected upon comparison of their mass fragmentation data with already known data of polyphenols from the same genus. The mass fragmentation data of the larger peaks were about 90% related to betulin and kamalachalcone C which are polyphenols and already known from the same genus, however the exact identification of the compounds were not focused in this study.

Distinct groups of compounds have been isolated from different parts of the plant; for example red compound [3], β-sitosterol, stigmasterol, bergenin, alpha–amyrin, 3′-prenylrubranine [16,17] and flavonoids such as kamalachalcones A, B [18]. A new flavanone, 4′-hydroxy isorottlerin, and two new chalcone derivatives, kamalachalcones C and D, isorottlerin, rottlerin and 5, 7-dihydroxy-8-methyl-6-prenylflavanone were isolated from red powder of glandular hair of *M. philippensis*[19]. Other compounds include phloroglucinol derivatives, mallotophilippens A and B, mallotophilippens C, D and E [9], 3-hydroxy-D:A-friedoolean-3-en-2-one [20], 2 α-hydroxy-D:A-friedooleanan-3-one and 3 α-hydroxy-D:A-friedoolean-an-2-one [21].

Rottlerin (5, 7-dihydroxy-2, 2-dimethyl-6-(2, 4, 6-trihydroxy-3-methyl-5-acetylbenzyl)-8-cinnamoyl-1,2-chromine) that is also called mallotoxin, is one of the major constituents of *M. philippensis*. Since the main peaks of the hexane root extract were unrelated to rottlerin, other bioactive compounds were responsible for the cytotoxic effect. It has been confirmed from the present study that hexane fraction is active against leukaemic cells and it is proposed that some potent anti-carcinogenic compounds exist in *M. philippensis* that warrant their identification.

## Conclusion

The results revealed that hexane soluble extract of *Mallotus philippensis* (Lam.) Muell.Arg. root possesses anti-leukaemic activity, which provide some mechanistic evidence for why indigenous people of Pakistan and other Asian countries found it useful for various ailments as well as food additive.

## Abbreviations

GC-MS: Gas chromatography mass spectrometry; MeOH: Methanol; EtOAc: Ethyl acetate; BuOH: n-butanol; MSD: Mass selective detector; EI: Electron ionization; HO: Hoechst 33258; PI: Propidium iodide; TBS: Tris base; PBS: Phosphate buffer.

## Competing interest

The authors declare that they have no competing interests.

## Authors’ contributions

MKD made significant contribution to perform various assays, acquisition and interpretation of data, conception and drafting of the manuscript. RAQ, MH, KM and RAK has made substantial contribution to conception and design, interpretation of data and drafting for intellectual content. All authors read and approved the final manuscript.

## References

[B1] Satyavati GV Gupta KA Tandon N Medicinal Plants of India Volume 2 1987 New Delhi: Indian Council of Medical Research 201 206

[B2] Gupta AK Chauhan JS Constituents from the stem of *Bauhinia variegate* Nat Acad Sci Lett 1984 7 15 16

[B3] Lounasmaa M Widen CJ Tuuf CM Huhtikangas A On the phloroglucinol derivatives of *Mallotus philippinensis* Planta Med 1975 28 16 31 1178784 10.1055/s-0028-1097825

[B4] Usmanghani K Saeed A Alam MT Indusynic Medicine 1997 Karachi: Research Institute of Indusyunic Medicine 285 287

[B5] Zabihullah O Rasheed A Akhter N Ethnobotanical survey in Kot Manzaray Baba valley Pakistan J Plant Sci 2006 12 115 121

[B6] Arisawa M Fujita A Hayashi T Morita N Kikuchi T Tezuka Y Studies on cytotoxic constituents in pericarps of *Mallotus japonicus* Chem Pharm Bull 1990 38 698 700 2347013 10.1248/cpb.38.698

[B7] Arisawa M Fujita A Morita N Koshimura S Cytotoxic and antitumor constituents in pericarps of *Mallotus japonicas* Planta Med 1990 56 377 379 2236292 10.1055/s-2006-960987

[B8] Fujita A Hayashi T Arisawa M Shimizu M Morita N Kikuchi T Tezuka Y Studies on cytotoxic constituents in pericarps of *Mallotus japonicas* J Nat Prod 1988 51 708 712 3210017 10.1021/np50058a007

[B9] Daikonya A Katsuki S Wu JB Kitanaka S Anti-allergic agents from natural sources Anti-allergic activity of new phloroglucinol derivatives from *Mallotus philippensis* (Euphorbiaceae) Chem Pharm Bull 2002 50 1566 1569 12499591 10.1248/cpb.50.1566

[B10] Zaidi SFH Yoshida I Butt F Yusuf MA Usmanghani K Kadowaki M Sugiyama T Potent bactericidal constituents from *Mallotus philippensis* against Clarithromycin and Metronidazole resistant strains of Japanese and Pakistani *Helicobacter pylori* Bio Pharma Bull 2009 32 631 636 10.1248/bpb.32.631 19336896

[B11] Nakane H Arisawa M Fujita A Koshimura S Ono K Inhibition of HIV-reverse transcriptase activity by some phloroglucinol derivatives FEBS Lett 1991 286 83 85 1713859 10.1016/0014-5793(91)80946-z

[B12] Jabbar A Raza MA Iqbal Z Khan MN An inventory of the ethnobotanicals used as anthelmintics in the Southern Punjab (Pakistan) J Ethnopharm 2006 108 152 154 10.1016/j.jep.2006.04.015 16730420

[B13] Kumar VP Chauhan NS Padh H Rajani M Non-timber forest products of Nepal J Ethnopharm 2006 107 182 188 10.1016/j.jep.2006.03.013 16678369

[B14] Tanaka R Nakata T Yamaguchi C Wada S Yamada T Tokuda H Potential anti-tumor-promoting activity of 3α-Hydroxy-D:A-friedooleanan-2-one from the stem bark of *Mallotus philippensis* Planta Med 2008 74 413 416 18484534 10.1055/s-2008-1034347

[B15] Grusch M Polgar D Gfatter S Leuhuber K Huettenbrenner S Leisser C Maintenance of ATP favours apoptosis over necrosis triggered by benzamide riboside Cell Death Diff 2002 9 169 178 10.1038/sj.cdd.4400937 11840167

[B16] Bandopandhyay M Dhingra VK Mukerjee SK Pardeshi NP Seshadri TR Triterpenoids and other components of *Mallotus philippensis*. Euphorbiaceae Phytochem 1972 11 1511

[B17] Ahluwalia VK Sharma ND Mittal B Gupata SR Novel prenylated flavanoids from *Mallotus philippensis* Muell Arg Ind J Chem 1988 27B 238 241

[B18] Tanaka T Ito T Iinuma M Takahashit Y Naganaw H Dimeric chalcone derivatives from *Mallotus philippensis* Phytochem 1998 48 1423 1427

[B19] Furusawa M Ido Y Tanaka T Ito T Nakaya K Ibrahim I Novel, complex flavonoids from *Mallotus philippensis* (Kamala tree) H Chim Act 2005 88 1048 1058

[B20] Kikuchi T Toyoda T Isolation and structure determination of pachysandiol-A and a note on the stereochemistry of cerin Tetrhedran Lett 1967 33 3181 3185

[B21] Talapatra SK Pradhan DK Talapatra B Terpenoids and related compounds: part XV. 3á-Hydroxyfriedel-2-one and 2â-acetoxyfriedel-3-one (epicerin acetate), two new pentacyclic triterpenoids from cork waste, their partial syntheses and one-step conversions to friedelin Ind J Chem 1978 16 361 365

